# Revisiting single cell analysis in forensic science

**DOI:** 10.1038/s41598-021-86271-6

**Published:** 2021-03-29

**Authors:** Davis R. L. Watkins, Dan Myers, Hannah E. Xavier, Michael A. Marciano

**Affiliations:** 1 Forensic and National Security Sciences Institute, Syracuse University, 100 College Place 120 Life Science Building, Syracuse, NY 13244 USA; 2 Fieldable Forensics, PO Box 141, Skaneateles Falls, NY 13153 USA

**Keywords:** PCR-based techniques, Molecular biology

## Abstract

Forensic science has yet to take full advantage of single cell analysis. Its greatest benefit is the ability to alleviate the challenges associated with DNA mixture analysis, which remains a significant hurdle in forensic science. Many of the factors that cause complexity in mixture interpretation are absent in single cell analyses—multiple contributors, varied levels of contribution, and allele masking. This study revisits single cell analyses in the context of forensic identification, introducing previously unseen depth to the characterization of data generated from single cells using a novel pipeline that includes recovery of single cells using the DEPArray NxT and amplification using the PowerPlex Fusion 6c kit with varied PCR cycles (29, 30, and 31). The resulting allelic signal was assessed using analytical thresholds of 10, 100, and 150RFU. The mean peak heights across the sample sets generally increased as cycle number increased, 75.0 ± 85.3, 147.1 ± 172.6, and 226.1 ± 298.2 RFU, for 29, 30, and 31 cycles, respectively. The average proportion of allele/locus dropout was most significantly impacted by changes in the detection threshold, whereas increases in PCR cycle number had less impact. Overall data quality improved notably when increasing PCR from 29 to 30 cycles, less improvement and more volatility was introduced at 31 cycles. The average random match probabilities for the 29, 30, and 31 cycle sets at 150RFU are 1 in 2.4 × 10^18^ ± 1.46 × 10^19^, 1 in 1.49 × 10^25^ ± 5.8 × 10^25^, and 1 in 1.83 × 10^24^ ± 8.09 × 10^24^, respectively. This demonstrates the current power of single cell analysis in removing the need for complex mixture analysis.

## Introduction

The interpretation of mixed DNA samples is a routine practice in forensic analyses, yet it often presents challenges. The challenges manifest when samples feature unequal contributions of donors, allele sharing, allelic drop-out, stutter, preferential amplification and stochastic effects, all of which can significantly influence the interpretation^1,2^. While the interpretation of mixtures with two contributors can be straightforward, the efficacy of interpretation—the ability to accurately infer specific contributor genotypes—of higher order mixtures is reduced as the contributor number increases^2–7^. These complex mixtures often cannot be interpreted with high confidence, which can lead to inconclusive results, poor resolution between donors or negatively affect the resulting likelihood ratios^3–7^.

An intuitive answer to reconcile the challenges associated with mixture interpretation is to analyze biological fluids from mixed samples separately. A classic example is the differential extraction procedure used to chemically separate sperm and epithelial cell (DNA) fractions in sexual assault cases^8^. However, this type of separation cannot discern multiple contributors from mixtures of one cell type or mixtures of non-sperm cells, e.g. white blood cells and epithelial cells. Single cell analyses can alleviate these complexities. Through targeted analysis of a single cell a single donor and a single source profile can be obtained from the cell of interest. Single cell analysis in forensic science is now possible given the increased sensitivity of human DNA amplification kits and new methods to recover single cells, namely the use of the DEPArray NxT system (Menarini Silicon Biosystems). Early single cell recovery methods include laser capture microdissection (LCM) and fluorescence-activated cell sorting (FACS). The pursuit of this type of technology in forensic science is not novel, Findlay et al. 1997 provides one of the first studies investigating forensic STR profiling of single epithelial cells. Cells were recovered through the use of “micromanipulation” procedures, likely using a type of microdissection. This study demonstrated that single cell processing using the forensic methodology at the time could obtain a full DNA profile in 50% (114/226) of single cells analyzed, an “acceptable” profile in 64% (144/226) of single cells, and yield detectable peaks in 91% (206/226) of the single cells analyzed^9^. LCM and FACS have inherent limitations including the lack of automatable procedures, low purity, reduced sensitivity and/or high minimum cell collection numbers^10,11^. More recently, the DEPArray system was introduced which uses a dielectrophoretic grid on a microfluidic device to capture, identify, select and subsequently recover the cell(s) of interest^12,13^. The utility of this method in forensic applications was previously demonstrated by Williamson et al.^13^ and Anslinger et al.^14,15^. Anslinger et al.^15^ used the DEPArray to separate single white blood cells and infer donor contributions from a mixture of blood from multiple contributors. Paired with the current human DNA amplification kits, the DEPArray mediated method can yield profiles from single cells that can assist in, or eliminate the need for, the currently used mixture deconvolution practices within the forensic community.

The most significant challenge facing the success of single cell analysis, and low template analysis in general, is the combination of the limited amount of template DNA (approximately 6.6 pg) and the sensitivity of the analytical methods. This leads to stochastic events that manifest as severe heterozygote imbalance and allele or locus dropout. Despite these limitations, Fontana et al.^12^ found that as few as 5 cells can provide approximately 78% of the expected profile. It is clear that ensuring sustained successful use of single cell analysis is contingent upon maximizing information content (maximizing signal strength and, in turn, minimizing allele and locus dropout). Strategies exist to further optimize the analysis of single cells, the most notable of which is increasing the number of PCR cycles; whereby increases in cycle number theoretically correspond to increases in total allelic product and decrease the occurrence of allele and locus dropout. The utility of this approach has been demonstrated by several studies^16–21^, where low template DNA (< 100 pg) has been shown to provide adequate genetic information when adjusting the PCR cycle number^21^. This suggests that increasing the PCR cycle number is a viable means of increasing signal (sensitivity) while simultaneously providing DNA data that is still interpretable. Cycle number increases may, however, introduce some confounding factors, e.g. heightened susceptibility to contamination and more drastic differences in inter- and intra-locus peak height balance^16,21–26^. Concerns are justified when analyzing 6.6 pg of DNA, as the resulting data is less predictable and more difficult to interpret than standard single source, multi-cell samples^27^. Thus, a balance must be struck between the amount of interpretable DNA data obtained and the number of PCR cycles.

The objective of this study is to characterize the expectations and behavior of single cells processed using a standard forensic workflow. We aim to: (1) investigate the feasibility of STR data analysis using single cells collected using the DEPArray NxT system, (2) assess the optimal PCR cycle number to maximize the information content of the analysis and minimize stochastic effects, and (3) offer insight into single cell reaction dynamics in a multiplex using inter- and intra- locus correlations specific to the PowerPlex Fusion 6c amplification kit.

## Results

### Peak height evaluation

The cycle set average peak heights increased as the cycle number increased with 29, 30, and 31 cycles resulting in mean peak heights of 75.0 ± 85.3, 147.1 ± 172.6, and 226.1 ± 298.2 RFU, respectively. A one-way ANOVA (α = 0.01) and post-hoc Tukey’s test (α = 0.05) where p < 0.001 demonstrated statistical significance between the mean peak heights across all cycle groups. The maximum average peak heights (across all loci in a given cycle set) also increase with cycle number—506RFU, 1825 RFU and 2358 RFU for the 29, 30 and 31 cycle sets, respectively).

**Figure 1:**
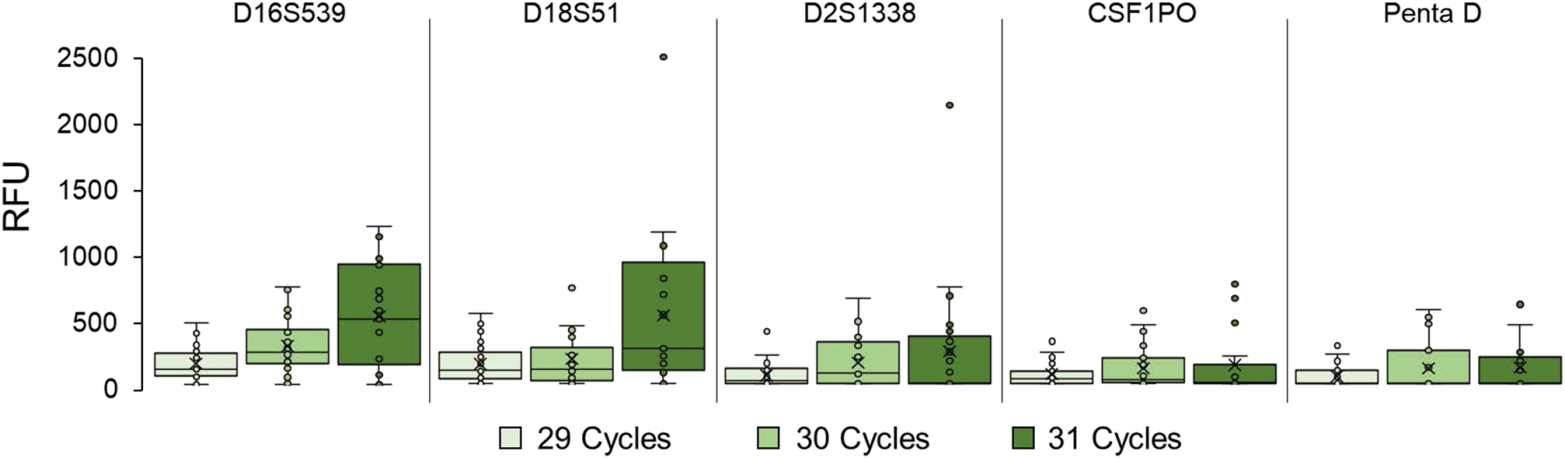
The distribution of locus-specific mean peak heights observed in 29, 30, and 31 cycle amplification groups in the green channel.

A more detailed, locus-specific, analysis of average peak heights shows the same trend, where average peak height (PCR product) increases within each locus as cycle number increases (Table 1, Fig. 1, Additional file 1-Figure S1). The overall mean peak height derived from the locus-specific average peak heights were 81.4 ± 42.8, 164.9 ± 100.3 and 253.8 ± 162.6 RFU for 29, 30 and 31 cycles, respectively. The range (minimum to maximum) and interquartile range also tend to increase as the cycle numbers increase, with ranges of 30–193 RFU, 57–511 RFU and 72–758 RFU for 29, 30 and 31 cycles, respectively. These values were calculated based on the presence of data above 10 RFU—these values represent the average peak heights when signal was detected and do not account for dropout.

**Table 1:** Locus-specific mean peak heights for single cells amplified using 29, 30 and 31 cycles and the PowerPlex Fusion 6c human DNA amplification kit.

Locus	Mean peak height (RFU)		
	29 cycles	30 cycles	31 cycles
AMEL	126.1 ± 104.5	300.7 ± 385.2	340.5 ± 335.9
D3S1358	115.4 ± 90.3	246.2 ± 164.3	249.0 ± 271.6
D1S1656	85.3 ± 75.9	226.3 ± 164.3	358.2 ± 329.0
D2S441	61.4 ± 63.9	116.6 ± 135.2	189.4 ± 211.0
D10S1248	88.5 ± 73.8	145.0 ± 150.0	277.4 ± 309.1
D13S317	55.6 ± 56.2	94.9 ± 115.0	176.6 ± 288.4
Penta E	41.6 ± 53.1	101.8 ± 148.0	122.6 ± 199.5
D16S539	152.5 ± 130.3	279.9 ± 196.5	493.9 ± 378.6
D18S51	142.4 ± 135.7	180.8 ± 208.9	493.8 ± 570.2
D2S1338	60.7 ± 83.4	154.7 ± 176.5	235.7 ± 462.2
CSF1PO	70.6 ± 90.0	113.3 ± 156.1	137.5 ± 242.8
Penta D	55.6 ± 74.8	111.3 ± 175.7	117.9 ± 194.9
TH01	50.6 ± 63.2	105.8 ± 106.2	158.6 ± 148.0
vWA	53.4 ± 60.6	77.0 ± 61.5	164.9 ± 176.7
D21S11	69.3 ± 60.6	124.0 ± 116.7	161.5 ± 183.0
D7S820	43.8 ± 46.1	100.5 ± 88.5	148.2 ± 154.5
D5S818	58.2 ± 65.4	99.3 ± 111.2	115.1 ± 151.3
TPOX	29.8 ± 42.5	69.6 ± 116.4	127.8 ± 234.2
D8S1179	50.9 ± 56.8	95.7 ± 61.1	186.6 ± 150.9
D12S391	100.5 ± 95.3	178.5 ± 153.8	266.0 ± 257.8
D19S433	55.4 ± 49.9	150.2 ± 152.4	171.7 ± 192.4
SE33	35.0 ± 41.2	59.0 ± 88.0	72.2 ± 129.3
D22S1045	33.3 ± 41.4	56.7 ± 80.3	75.7 ± 123.1
DYS391	192.5 ± 160.8	511.3 ± 106.6	757.6 ± 450.2
FGA	116.8 ± 100.9	234.7 ± 175.1	353.5 ± 319.9
DYS576	103.3 ± 75.0	281.8 ± 235.4	498.2 ± 285.4
DYS570	150.6 ± 85.8	237.3 ± 109.6	401.8 ± 369.5

**Figure 2:**
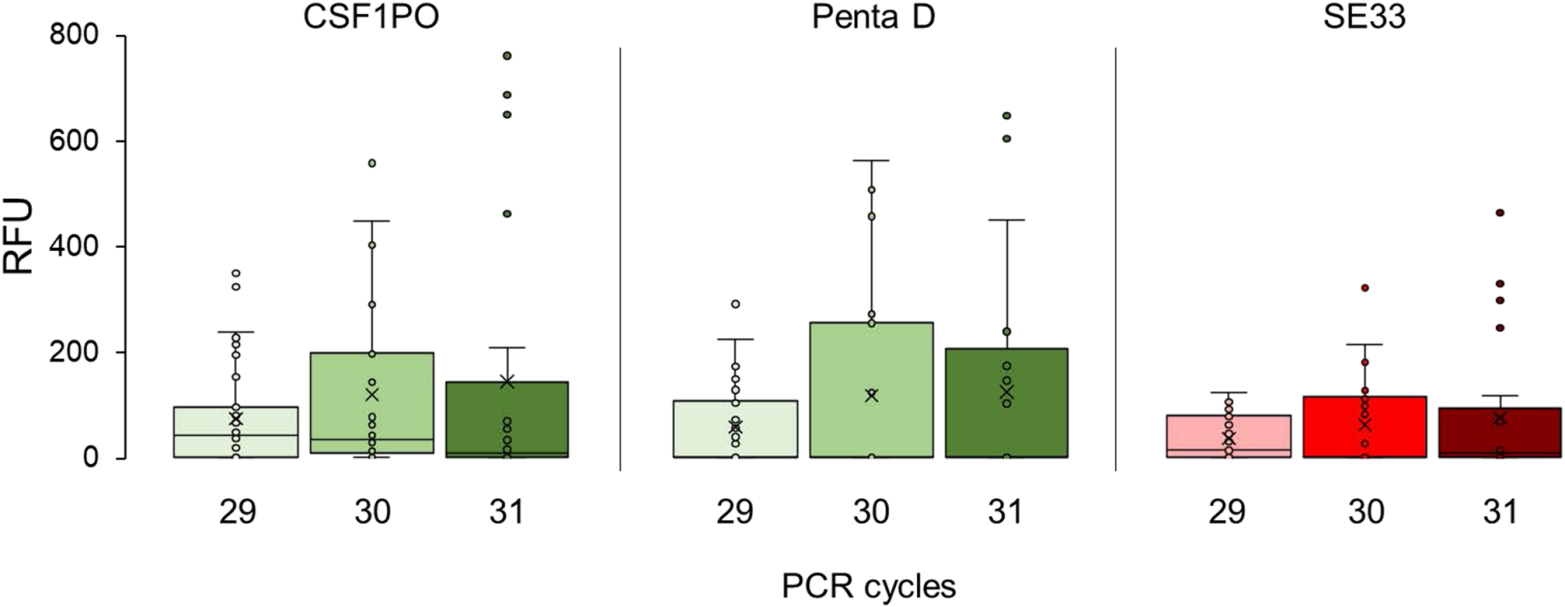
Locus-specific peak heights (RFU) vs. cycle number for the CSF1PO, Penta D, and SE33 loci amplified using the Promega PowerPlex Fusion 6c kit.

The distribution of peak heights in the 29 cycle set is consistently tighter than the 30 and 31 cycle groups. Generally, the highest mean peak heights (locus-specific) across the varying cycles are found in the 31-cycle sample set, however this sample set also displays the widest range of peak heights, thus greater volatility (Fig. 2, Additional file 1—Figure S1). Although the maximum peak heights were observed most commonly in individual samples in the 31 cycle set, the mean peak heights in the 30 and 31 cycle sets were often similar and, in some cases, higher in the 30 cycle set e.g. CSF1PO, Penta D and SE33 (Fig. 2).

### Proportion of allele and locus dropout

Measures of allele and locus dropout across the varied cycle numbers (29–31) and analytical thresholds (10, 100, and 150 RFU) were used to assess the success of single cell analyses. Complete profile dropout was uncommon, occurring in 3 of 38 samples in the 29 cycle-100 RFU set, 8 of 38 samples when the analytical threshold was increased to 150 RFU, and in only one of 21 samples in the 31-cycle 150 RFU set. Profile dropout (complete loss of allelic information) was *not* observed in the 30 and 31-cycle 100RFU and 30-cycle 150 RFU set.

The cycle set mean proportion of allele dropout and locus dropout were consistent with the expected results, where increasing the threshold led to increased proportions of dropout (Table 2). When comparing the cycle set proportion of dropout within threshold groups the mean proportions of allele and locus dropout remain fairly static with slight decreases as cycle number increases (Table 2). An analysis of variance (ANOVA) indicates that the average proportion of allele and locus dropout across cycle numbers at 10RFU (p = 0.688 and p = 0.458, respectively) and 100 RFU (p = 0.129 and p = 0.111, respectively) are not significantly different (α = 0.99). In contrast, we observed a significant difference in the cycle number specific dropout proportions using an analytical threshold of 150RFU (p = 0.0096 and p = 0.0073, respectively). A post-hoc Tukey test (α = 0.05) showed the level of allele dropout observed in the 29 cycle sample set is significantly higher than the 30 and 31 cycle sample sets (p = 0.002), whereas the 30 and 31 cycle sets are not significantly different. Similarly, no significant difference was observed between the level of locus dropout among the 29 and 30 and the 30 and 31 cycle sets (ANOVA α = 0.01, p = 0.007, Tukey’s test α = 0.05, p = 0.01).

**Table 2:** Summary of the mean cycle set allele and locus dropout, the range (minimum-to maximum values observed separated by cycle number and the analytical threshold value used to assess the signal) and the p values of the ANOVA comparing the variance at each threshold across cycle numbers at α = 0.99.

Threshold (RFU)	Cycle #	n	Proportion samples w/full dropout	Allele dropout			Locus dropout		
				Average	Range	p value	Average	Range	p value
10	29	38	0/38	0.405 ± 0.223	0.044–0.791	0.688	0.257 ± 0.194	0.00–0.667	0.458
	30	22	0/22	0.369 ± 0.199	0.022–0.744		0.233 ± 0.191	0.00–0.667	
	31	21	0/21	0.429 ± 0.269	0.044–0.954		0.313 ± 0.277	0.00–0.917	
100	29	38	1/38	0.675 ± 0.280	0.154–1.00	0.129	0.579 ± 0.320	0.00–1.00	0.111
	30	22	0/22	0.550 ± 0.289	0.044–0.977		0.429 ± 0.309	0.044–0.958	
	31	21	0/21	0.538 ± 0.294	0.044–0.977		0.430 ± 0.311	0.044–0.958	
150	29	38	7/38	0.788 ± 0.234	0.154–1.00	0.0096	0.712 ± 0.287	0.00–1.00	0.0073
	30	22	0/22	0.619 ± 0.299	0.044–0.977		0.508 ± 0.330	0.00–0.958	
	31	21	1/21	0.581 ± 0.300	0.044–1.00		0.474 ± 0.322	0.00–1.00	

**Figure 3:**
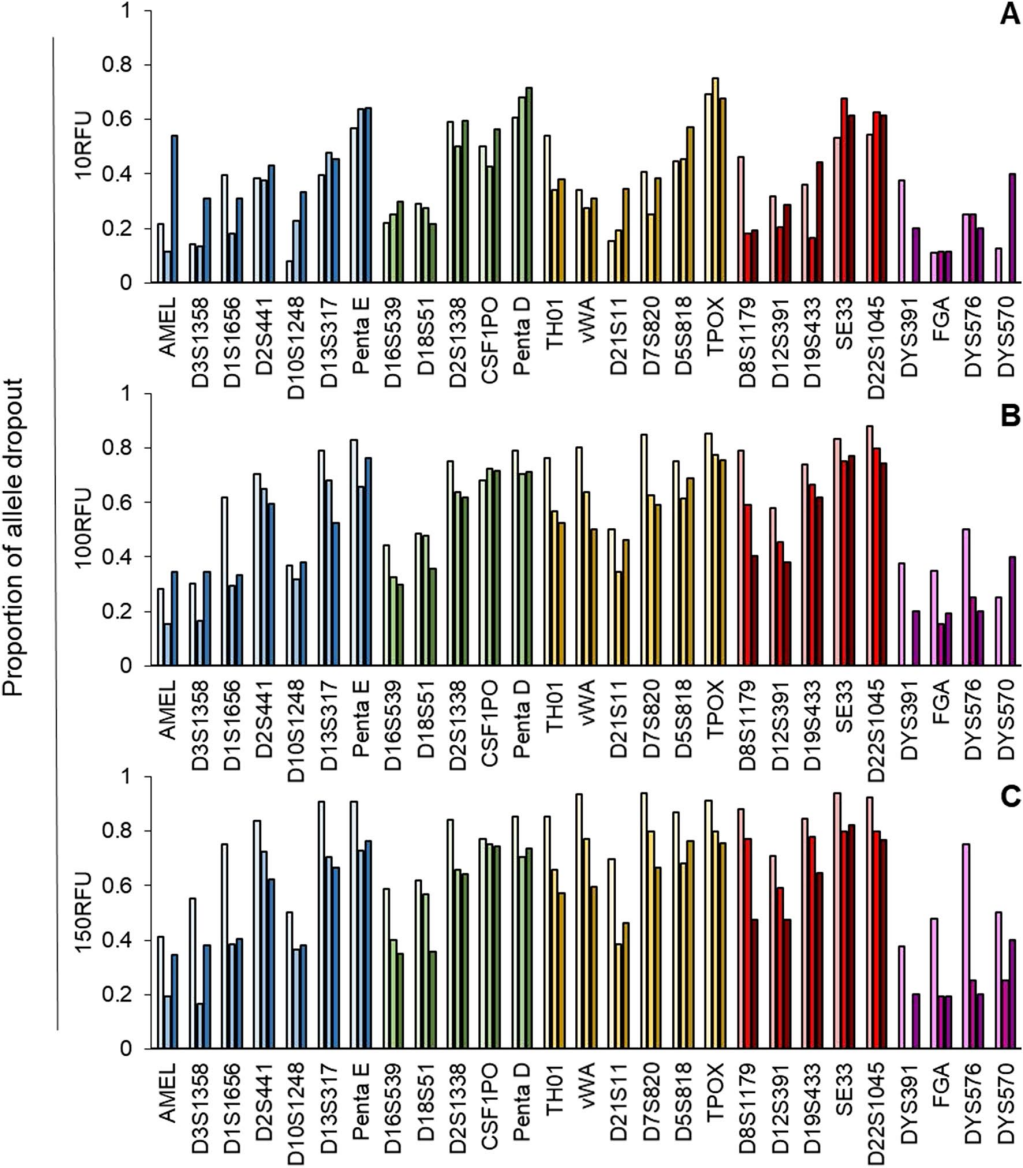
Average proportions of allele dropout by locus for single cells at 29, 30 and 31 cycles using the PowerPlex Fusion 6c kit analyzed at (**a**) 10 RFU, (**b**) 100RFU and (**c**) 150RFU. Cycle numbers within each locus from left to right are 29, 30, and 31, respectively and the colors (blue, green, yellow, red and purple) represent the dye channel.

**Figure 4:**
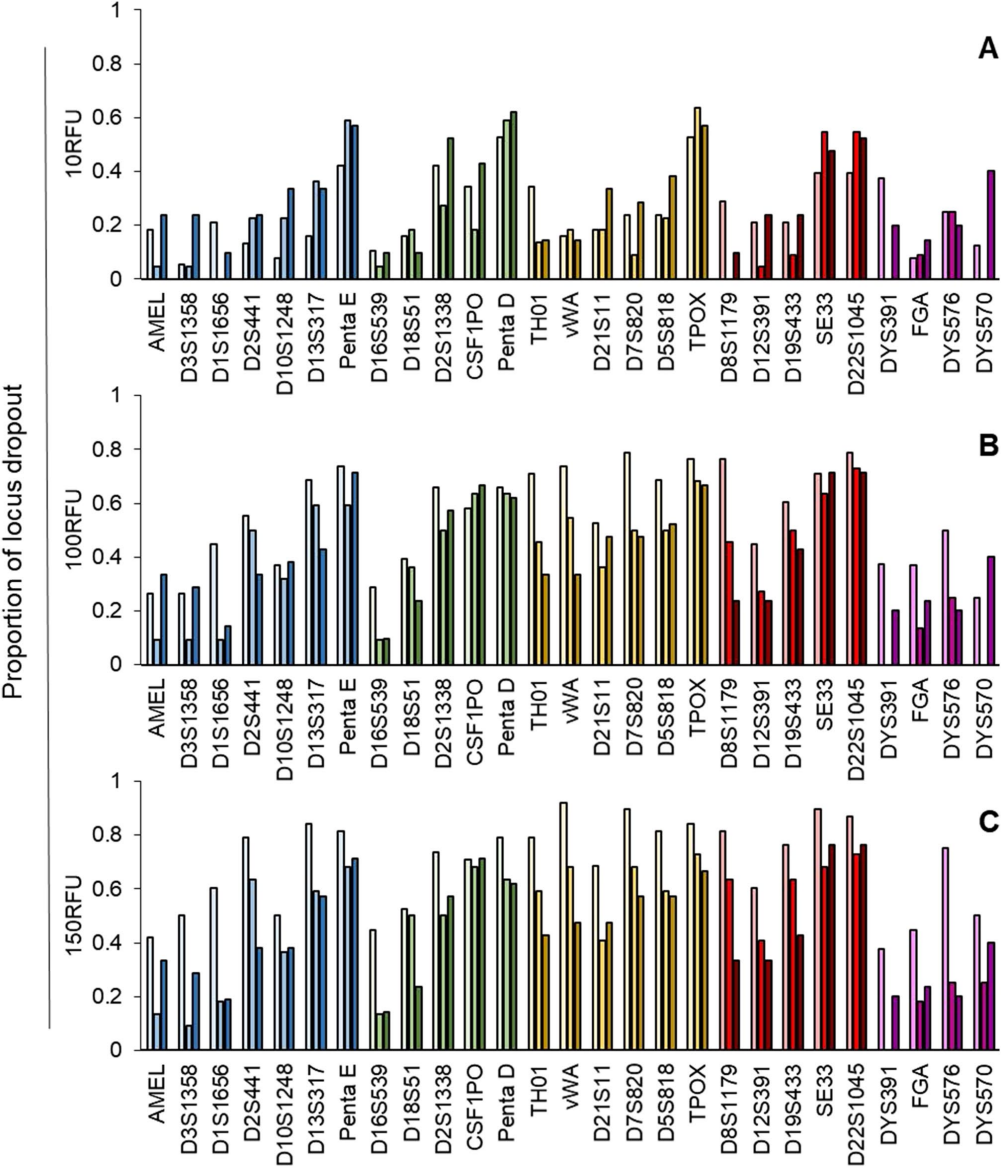
Average proportions of locus dropout by locus for single cells at 29, 30 and 31 cycles using the PowerPlex Fusion 6c kit analyzed at (**a**) 10 RFU, (**b**) 100RFU and (**c**) 150RFU. Cycle numbers within each locus from left to right are 29, 30, and 31, respectively and the colors (blue, green, yellow, red and purple) represent the dye channel.

The level of dropout observed in locus-specific sets have a direct relationship with the analytical threshold, a trend that was also observed in the cycle-specific data (where all dropout proportions were pooled and averaged) (Figs. 3, 4). Thus a clear pattern emerges in both the allele and locus dropout analyses. As expected, the proportions of dropout increase as the size of the locus increases (Fig. 3, Additional file 1—Table S3, Fig. 4, Additional file 1—Table S4). Increases in the cycle number generally lead to decreased proportions of allele dropout, which is most apparent in the 150RFU analyses (Fig. 3, Additional file 1—Table S3). This trend is less apparent in the 10RFU threshold group, where 18 of the 27 loci (66.7%) show higher levels of allele dropout at 31 cycles compared to the 29 cycle. Whereas the sample sets analyzed at 100 RFU and 150 RFU show this relationship in five and two of the loci, respectively (Fig. 3, Table S3). Amelogenin and DYS570 share this relationship across all thresholds used.

Locus-specific locus dropout behaves in a similar manner across cycle numbers where the 100 and 150 RFU groups tend to decrease as cycle number increases (Fig. 4, Additional file 1—Table S4). The relationship of locus dropout and cycle number in the 10 RFU group largely mirrors that of the proportion of allele dropout, where 19 of 27 loci have higher levels of locus dropout at 31 cycles compared to the 29-cycle group (Fig. 4, Additional file 1—Table S4). In many cases, both the proportions of locus-specific allele and locus dropout at 30 and 31 cycles are similar, indicating that a stochastic threshold may be reached at 30 cycles, where increasing the cycle number beyond 30 cycles does not increase the information content of the sample.

### Measuring success of single cell analysis

Allelic dropout and heterozygote imbalance are inevitable consequences of analyzing low template DNA samples. Therefore, robust characterization (establishing expectations) of the performance of single cell analyses is critical to the success and potential implementation of this method. The RMP was used to evaluate the rarity of the DNA profiles obtained from single cell analysis using 29, 30 and 31 cycles and 100 and 150RFU analytical thresholds. The average RMP for the 100 RFU analysis were 1 in 1.2 × 10^23^ ± 7.04 × 10^23^, 1 in 1.94 × 10^28^ ± 8.9 × 10^28^, and 1 in 5.76 × 10^26^ ± 1.82 × 10^27^, for 29, 30 and 31 cycles, respectively with medians of 2.74 × 10^4^, 6.36 × 10^8^ and 8.90 × 10^11^. The data analyzed using a 150 RFU analytical threshold yielded RMPs of 1 in 2.4 × 10^18^ ± 1.46 × 10^19^, 1 in 1.49 × 10^25^ ± 5.8 × 10^25^, and 1 in 1.83 × 10^24^ ± 8.09 × 10^24^, for 29, 30, and 31 cycles, respectively (Table 3), with medians of 1.22 × 10^2^, 8.04 × 10^6^ and 4.60 × 10^9^. Average RMPs decreases when the number of cycles was increased from 29 to 30, whereas a decrease was observed when increasing PCR cycles from 30 to 31. And, as expected, average RMPs increased when the analytical threshold was increased from 100 to 150RFU.

**Table 3:** Random match probabilities (calculated without theta corrections and the use of the “2p” rule) for profiles generated from single cells amplified with the PowerPlex Fusion 6c amplification kit using 29, 30, and 31 cycles and analyzed using analytical thresholds of 100 and 150RFU.

Cycle number	AT = 100RFU		AT = 150RFU	
	Mean RMP	RMP range	Mean RMP	RMP range
29	1.20 × 10^23^ ± 7.04 × 10^23^	0–4.34 × 10^24^	2.40 × 10^18^ ± 1.46 × 10^19^	0–8.99 × 10^19^
30	1.94 × 10^28^ ± 8.90 × 10^28^	23.5–4.18 × 10^29^	1.49 × 10^25^ ± 5.8 × 10^25^	15.7–2.68 × 10^26^
31	5.76 × 10^26^ ± 1.82 × 10^27^	8.87–6.10 × 10^27^	1.83 × 10^24^ ± 8.09 × 10^24^	0–3.71 × 10^25^

*AT* analytical threshold.

Contamination or allele drop-in may become more likely given an increased number of PCR cycles. However, there were no instances of allele drop-in across 81 samples, and only one sample was contaminated (this sample was excluded from analysis). The contaminated profile was generated from a sample comprised of a single epithelial cell from a known female donor and amplified using 31 cycles. The sample was thus a mixture between the known (expected) profile and an unknown male—not attributable to any laboratory personnel. In addition, allelic signal was not detected in any of the extraction and PCR negative controls across the entire study. The unknown male profile was present at 18 loci and an average peak height of 884.4 ± 438.3 (at 5 loci that the unknown male clearly did not share with the known).

### Inter-locus comparisons

The relationship among the loci was assessed through (1) a correlation of total allelic product, and (2) the individual locus pairs in obtaining detectable signal across analytical thresholds and cycle number variants. These assessments are kit dependent and therefore represent the amplification dynamics of single cells amplified using the PowerPlex Fusion 6c amplification kit with 29, 30, and 31 PCR cycles.

**Figure 5:**
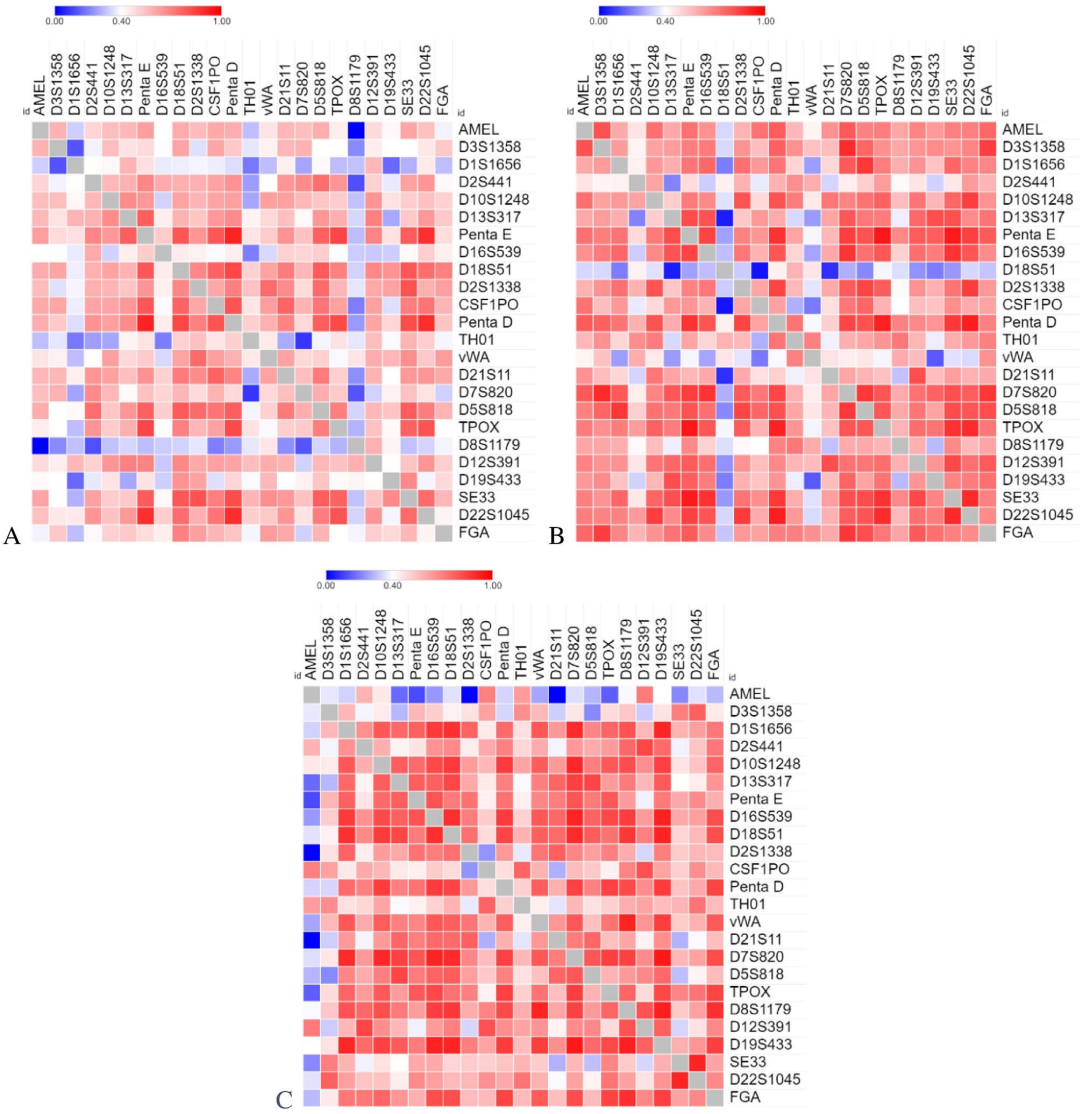
Spearman correlation of total allelic product at 29 cycles (**a**), 30 cycles (**b**), and 31 cycles (**c**) where the value of the correlation coefficients are: very high (0.9–1.0), high (0.7–0.89), moderate (0.4–0.69), low (0.1–0.39), and negligible (0.0–0.1).

The average correlation coefficients across loci of the total allelic product generally increased from 29 to 30 cycles and remained consistent between 30 and 31 cycles − 0.52 ± 0.09, 0.61 ± 0.11, 0.62 ± 0.1, for 29, 30 and 31 cycles, respectively (Fig. 5). The weakest correlations were observed across the 29-cycle set, whereas the 30 and 31 cycle sets showed similar correlations with the 30-cycle set being most consistent across loci. The relationships between the loci remain largely positive, with only slightly negative values (− 0.004) observed between the Amelogenin and D8S1179 loci (and only in the 29 and 31 cycle sample sets). In general, patterns emerge where the total allelic product of smaller loci are correlated with larger loci and large loci are correlated to other large loci. Several individual locus correlations are also particularly noteworthy. D7S820 and D5S818 exhibit high to very high correlation with most loci at 30 and 31 cycles. TPOX remains highly correlated with Penta E, Penta D, and D22S1045 and is consistently correlated at moderate to high with FGA across 29, 30, and 31 cycles. D22S1045 and SE33 are highly to very highly correlated with one another across cycle sets. Penta E is highly correlated to D13S317. vWA is consistently correlated with D8S1179 at low to high levels. D7S820 is very highly correlated with D19S433, D18S51, D16S539 and D1S1656. In contrast, TH01 and D2S441 consistently display a low to moderate correlation across all samples and cycle numbers. D8S1179, D7S820, D19S433, D16S539 and D1S1656 exhibit stepwise increases in correlation with most of the remaining other loci as cycle number is increased. Interestingly, correlation coefficients at D18S51 and vWA are consistently low in the 30-cycle sample set compared to the 29 and 31 cycle sets (Fig. 5, Additional file 1—Figure S5, Additional file 1—Table S5).

## Discussion

The use of single cell analyses (one cell–one contributor) in concert with tools such as the DEPArray NxT in combination with the DEPArray LysePrep kit has the potential to revolutionize forensic DNA analysis. This workflow provides a means to reduce and/or eliminate mixtures through the selective separation and recovery of specific cells or cell types. This, or other single cell recovery methods, can be inserted into a forensic workflow without altering the underlying methods, e.g. DNA extraction, quantification, amplification and detection. The data indicate that single cell analysis using the pipeline described herein with 30 PCR cycles will produce ample information content to achieve average RMPs of 1 in 1.94 × 10^28^ ± 8.9 × 10^28^. This analysis can provide unprecedented granularity, often permitting the complete removal of the need for mixture analyses. It will also allow for more informed conditioning (“subtraction”) of known contributors (donor of the typed single cell) from complex mixtures, significantly augmenting the chance of deconvolution. In turn, this will provide analysts the ability to make more robust interpretations, leading to more informative comparisons and more probative likelihood ratios.

Single cell analysis has a cost–benefit relationship where one will have to weigh the potential information content provided by a single cell against that of mixture interpretation. The benefits are realized when formulating the assumptions. The most obvious and significant assumption is the accurate inference of a single contributors, provided by the isolation of single cells (assumption 1). This acts to constrain subsequent assumptions regarding heterozygote balance and stochastic events. Assumption 2 relates to heterozygote peak height balance. Since the totality of signal is the result of a single donor, two alleles at any given locus can be considered a homologous pair, even when significant peak height disparity exists. This is in contrast to multi-cell or mixture analyses where the presence of severe peak height imbalance may indicate more than one contributor where it may be more difficult or impossible to make an accurate conclusion regarding the allele pairs. Assumption 3 relates to the presence of allele dropout—stochastic thresholds become unnecessary because if a single allele is detected above the analytical threshold it can be assigned to an individual donor, regardless of the individual’s zygosity at the locus. Routinely, mixture interpretation does not accommodate for this assumption, particularly if more than one low level contributor is present. Ultimately, implementation of single cell analyses will require a quantitative evaluation of the expectations and limitations of the pipeline, specifically in evaluating the impact of stochastic events.

The success of single cell analysis will largely be based on the level of stochastic events observed, namely allele and locus dropout. We examined methods to decrease the level of dropout that included increasing the number of PCR cycles stepwise from 29 to 31 and applying different analytical thresholds at 10, 100, and 150 RFU. The use of the 100 and 150 RFU analytical thresholds were used to mirror the process a laboratory may use, whereas the 10 RFU threshold was used to demonstrate the detectable limit—was signal present above baseline (true zero). The process described in the study, DEPArray NxT-LysePrep extraction-PowerPlex Fusion 6c, yields a baseline with low levels of noise, thus permitting differentiation of signal from noise at lower analytical thresholds (data not shown). We expect the frequency of dropout to increase with increasing thresholds, whereas the cycle number and level of dropout will have an inverse relationship (increasing the cycle number leads to increased amounts of PCR product and thus higher peak heights and potentially lower levels of dropout).

### Sensitivity

#### Peak height

The peak intensity/detection is a primary concern associated with low template samples. Increasing the PCR cycle number is a common strategy to increase signal and the results met expectations. Statistically significant increases in mean peak heights were observed as the cycle number increased from 29 to 31. The 30 and 31 cycle sets exhibit mean peak heights approximately at or above 150 RFU (30 cycles—mean peak height 147RFU) with minima greater than 50RFU. This is consistent with previous studies that demonstrated a mean peak height of 203 RFU^12^. Since many laboratories use analytical thresholds in the 100–150 RFU range, the results indicate that single cell separation with the DEPArray can provide significant signal to be practical for forensic laboratories. In addition, baseline noise is exceedingly low, which may permit laboratories to lower thresholds to further increase information content. We hypothesize that this is a result of the DEPArray-mediated purification where cells are purified from potential contaminates in the sample. In addition, the use of stochastic thresholds become significantly less critical, owing to the profile resulting from a single donor.

When examining locus-specific dynamics, the mean locus-specific peak heights increase as cycle number increases, an expected result of increased cycle numbers. However, the range and interquartile range generally increased with cycle number, indicating more variation in the allelic signal, i.e., stochastic events become both more common and more substantial as the cycle number increased. Moreover, although peak heights greater than 750 RFU were observed in the 31 cycle set, greater volatility is present where dropout may also occur. In some instances, the 30 cycle set exhibited greater locus-specific mean peak heights than those in the 31-cycle set. This supports the hypothesis that a stochastic limit has been reached, indicating that little benefit would be obtained by increasing the cycle number beyond 30 cycles.

#### Allele and locus dropout

The level of dropout (allele and locus) becomes more substantial as the quantity of template DNA decreases, thus, it will be common when analyzing a single cell (6.6 pg of DNA). Presenting both metrics permits a quantitative examination of the results that one would expect when analyzing single cells. Variables such as the analytical threshold and the number of cycles will influence the levels of dropout observed, where increasing thresholds are expected to increase dropout and increasing PCR cycles are expected to decrease the frequency of dropout. The cycle set mean proportion of allele and locus dropout increased with increasing thresholds, holding true across the samples amplified using 29, 30, and 31 cycles. Interestingly, the dropout levels remained relatively static when amplification cycles were increased and analytical thresholds remained constant. One would expect the frequency of dropout to decrease as cycle number increases, we observed that the levels remain somewhat static and not significantly different between the 10 and 100 RFU groups. The only significant decrease in allele and locus dropout was observed between the 29 and 31 cycle samples analyzed using a 150RFU analytical threshold (average proportion of dropout is higher in 29 than 31). This result is consistent with expectations when cycle numbers are increased. When considering the range of both allele and locus dropout, the 30 cycle group remains largely equivalent to, or slightly lower than, the 29 and 31 cycle ranges. This indicates that the most robust results, i.e., the highest allelic information content, are obtained using 30 cycles. This further supports our hypothesis that a stochastic limit has been reached, where increasing the cycle number beyond 30 cycles does not impart a significantly more advantageous detection sensitivity.

### Evaluating the expected success of single cell analysis

Successful execution of this pipeline is dependent on two factors: the ability to successfully recover intact cells and the quality of the DNA. The DEPArray was previously used to recover cells from a variety of sample types that mimic the type or quality of samples found at a scene—15-year-old proficiency test samples (stored at − 20 °C), post-coital sperm and epithelial cells and cells dried at room temperature^13^. This method will prove useful in the field because many scenes are processed soon after the crime and proper sample collection and storage methods are used to maintain the integrity of the biological samples. Further work is currently underway to investigate the efficacy of this method on lower quality samples, e.g., samples that are significantly older and degraded.

Random match probabilities were used to demonstrate the rarity of the profiles obtained when analyzing single cells. Although these calculations are dependent on the expected alleles from the individual donors used in this study, this measure does provide a general expectation of the value of the profiles obtained when performing single cell analysis. Average RMPs increased with increases in the AT, a result that would be expected considering the potential information loss that can occur when increasing thresholds. However, even the most restrictive analysis parameters (29 cycles and AT = 150RFU) yielded an average RMP of greater than 1 in 1 × 10^18^ (or 1 in one quintillion). The results indicate, again, that 30 PCR cycles, regardless of AT, produces the most discriminating results and avoids potential deleterious effects of further increases in cycle number. And, while the ranges of the RMPs in the 30 cycle set vary from 23.5 and 4.18 × 10^29^ (AT = 100RFU) and 15.7 to 2.68 × 10^26^ (AT = 150RFU), the median values are 6.36 × 10^8^ and 8.04 × 10^6^, for 100 and 150RFU analyses, respectively. This indicates that over 50% of the time RMPs of less than 1 in 100 million or 1 in one million would be expected using 100 or 150RFU thresholds, respectively. Although these values may not be what forensic scientists are accustomed to, one must consider that these were generated using a single cell where no mixture is present. These RMPs may represent significantly more discriminating results than one would expect when deconvoluting a low-level contributor from a mixture.

The ability to isolate single cells provides a further understanding of downstream methods such as the amplification dynamics of the PowerPlex Fusion 6c DNA amplification kit. The correlation of the total allelic product across the loci will help to inform the analyst which loci can be expected to “co-amplify” successfully. The average correlations across cycle sets largely mirrored expectations, where increased cycles led to increased signal and thus a stronger correlation. The strongest correlations were between small and large loci and large loci with other large loci. This result is intuitive; smaller loci are typically preferentially amplified, meaning that if larger loci have been detected, the smaller loci are expected to also be present. Similarly, if a given large locus yields detectable signal then it is reasonable to expect that some other larger loci also exhibit detectable signal. The strong correlation that D7S820 and D5S818 exhibit with most other loci indicate that these loci can be used as a benchmark (i.e., if these loci are not present, useable results are unlikely). This can potentially be used to detect sample inhibition or exceedingly low DNA template amounts. Other significant indicator loci are: (1) TPOX—an indicator locus for medium/large loci such as Penta E, Penta D, D22S1045, and FGA, and (2) D7S820, which is strongly correlated with D19S433, D18S51, D16S539, and D1S1656. The strength of these findings are supported by consistent correlations across samples within and between the varied PCR cycle number sets. Therefore, these relationships are likely valid. Other loci were less consistent, namely D8S1179, vWA, D18S51, D19S433, D16S539, and D1S1656. The lack of consistency can likely be explained by single cell (low template) stochastic events; therefore, we cannot completely discount the potential value of these loci as indicators. Again, we observe the 30-cycle sample set provides the most consistent correlations across loci with an average similar to the 31-cycle sample set, supporting the recommendation to use 30 PCR cycles when amplifying approximately 6.6 pg of DNA.

Contamination was identified in sample amplified using 31 PCR cycles. This was isolated to a single sample, as all other samples were single source and all reagent blanks and PCR negative controls were free of allelic signal. The contaminating profile was clearly from a low-level source, a single cell, or cell-free DNA as the profile demonstrated significant stochastic effects. The known epithelial cell was recovered from a DEPArray NxT run of a single known female donor. Therefore, it is likely that the contamination did not occur during the recovery phase. The contaminate likely entered the pipeline during extraction or amplification because the deduced donor did not match any individual that works in the laboratory. The unknown male DNA was likely deposited on a tube, pipette tip, or other consumable prior to entering the laboratory. Alternatively, it is possible that dust particles entered into a consumable at some point during the analyses. This was compounded by the use of 31 cycles, where it is possible to increase signal of trace amounts of DNA. Ultimately, in practice, the identification of contamination is straightforward when single source samples (single cell) are routinely expected. This event was isolated to one sample across the study; therefore, it is expected that the risk in practical settings will be equally as rare.

### Cycle number optimization

The data presented in this study strongly supports the use of 30 amplification cycles as opposed to 29 and 31 cycles. 30 cycles provide the most consistent peak heights with low proportions of allele and locus dropout. The 29 cycle set exhibited the highest levels of allele and locus dropout when using 100 and 150 RFU analytical thresholds (Table 2). One would expect that the proportion of allele and locus dropout would increase from 30 to 31, however there was no statistical difference between the levels of dropout observed between the two sets. This holds true even when increasing the analytical thresholds, where the level of dropout is expected to increase more significantly as threshold is increased for the samples amplified at a lower cycle (30). The differences between allele and locus dropout in the 30 and 31 cycle sets in the 100 RFU analysis were only 0.012 and − 0.001, respectively and 0.038 and 0.034, respectively, in the 150 RFU analysis. The average peak heights across the cycle sets adhered to expectations, where peak heights increased as cycle numbers increased. However, the 30 cycle sample set exhibited average cycle-wide mean peak heights of 147.1 ± 172.6 RFU and a locus specific average of 164.9 ± 100.3 RFU. These are values that will be at or above the analytical threshold used by many laboratories. The use of 31 cycles does lead to higher peak heights than observed in the samples amplified using 30 cycles. However, there is no change in the level of dropout observed, more pronounced stochastic effects are present, and there is an added risk of detecting drop-in alleles. Overall, the use of 30 cycles is recommended because it provides more predictable results with acceptable peak heights and levels of dropout to obtain single source profiles with an average RMP of less than 1.49 × 10^25^.

## Conclusion

This study provides a comprehensive characterization of the behavior of single cells in a forensic pipeline and, in doing so, strengthens support for the use of single cell analyses. Implementation of this pipeline will alleviate many of the complexities associated with mixture analysis, including but not limited to, simplifying data interpretation, improving overall laboratory efficiency, and increasing the effectiveness of downstream statistical analyses. However, the decision to use single cell data and the complexities associated with this type of analysis must be weighed against similar challenges in interpreting complex mixtures. The path to implementation, as laid out in this study, requires minimal changes to the standard forensic DNA analytical cascade, namely: (1) the use of the DEPArray NxT for cell separation and recovery and (2) the use of 30 amplification cycles rather than 28–29 amplification cycles. This results in profiles generated from single cells that exhibit an average RMP of 1 in 1.49 × 10^25^, when using an analytical threshold of 150 RFU.

## Methods

This study revisits single cell analyses using a novel pipeline that includes isolation and recovery of single cells using the DEPArray NxT and amplification using the PowerPlex Fusion 6c kit with varied numbers of PCR cycles (29, 30, and 31); the resulting allelic signal was assessed using analytical thresholds of 10, 100 and 150RFU.

### Samples

Biological samples were collected from two sources: (1) internally-generated buccal epithelial cell samples collected using a cotton swab from a male and female volunteer, and (2) mock mixture samples provided by Menarini Silicon Biosystems (MSB). The MSB samples consisted of the following body fluids combined on a COPAN flocked swab: saliva/semen, saliva/blood, semen/blood and saliva/semen/blood. Samples were either processed immediately or stored at 4 °C. This study was approved by the Syracuse University Institutional Review Board, all samples were stripped of identifying information, all experiments were performed in accordance with relevant named guidelines and regulations and informed consent was obtained from all participants and/or their legal guardians.

### Sample processing and analysis

All samples were processed using the Menarini Silicon Biosystems DEPArray Forensic Sample Prep Kit (REF FORSE). This workflow permits staining different cell types using stain-antibody conjugates for white blood cells (Phycoerythrin) and epithelial cells (Allophycocyanin), as well as a nucleic acid stain (DAPI: 4′,6-diamidino-2-phenylindole). Sperm cells were not targeted in this study; a previous study examined the analyses of single sperm cells^13^. The stained and purified samples were subsequently run on the DEPArray NxT using the forensic protocol where single cells were identified and recovered. In total, 64 single epithelial cells and 17 white blood cells were recovered across seven instrument runs. Each cell then underwent DNA extraction using the DEPArray LysePrep Kit (REF DALYS), where final reaction volumes totaled 5-7µL, avoiding centrifugal concertation methods. This extraction protocol permits direct addition of amplification reagents to the extraction tube, avoiding tube transfers.

All samples were amplified on the Life Technologies Veriti thermal cycler using the Promega PowerPlex Fusion 6c human DNA amplification kit (DC2705). Amplification was performed using half reactions (12.5 µL total reaction volume). Manufacturer recommended cycling parameters were used; however, cycle numbers varied stepwise from 29 to 31 cycles where 38 single cells were amplified using 29 cycles, 22 cells were amplified with 30 cycles, and 21 single cells were amplified using 31 cycles. Fragment analysis was performed on the ThermoFisher Scientific 3500xL Genetic Analyzer (POP-4, 36 cm capillary array) using the manufacturer’s recommended injection protocol; subsequent software analyses were performed using OSIRIS 2.12.1 (Open Source Independent Review and Interpretation System)^28^.

Single cells were first analyzed by assessing the level of allelic signal (RFU) detected at each locus across 29-, 30- and 31-PCR cycle sample sets at thresholds of 10, 100, and 150RFU. These values were chosen due to widespread use of analytical thresholds between 100 and 150RFU; the 10 RFU threshold is used to demonstrate the maximum information gain. It is not uncommon that instruments would yield a threshold of between 15 and 20RFU when calculating analytical thresholds using mean + 3 standard deviations (a 99% confidence interval)^29^. Average peak heights at each autosomal locus was calculated as described in^13^, where the peak heights of heterozygotic loci were averaged and the peak heights of homozygous loci were divided by two. The average allelic product of a heterozygous locus where allelic dropout occurred was calculated by dividing the detected peak by 2, where the peak that dropped out has an RFU value of 0. Peak heights of the haploid Y-STR loci were not divided. The mean peak heights across the samples within a specific cycle set were calculated by taking an average of the allelic product observed at each cycle number. This was then statistically assessed using a one-way ANOVA (α = 0.01) and the post-hoc Tukey’s test (α = 0.05). Similarly, locus-specific mean peak heights were calculated by averaging the allelic product at each locus.

The proportion of allele and locus dropout were calculated (1) in a cycle specific manner, where the number of alleles lost in each sample within a cycle set were summed and divided by the total number of expected alleles and (2) in a locus-specific manner where the number of alleles lost at a locus were summed and divided by the number of expected alleles. The proportion of cycle set allele and locus dropout was statistically assessed using a one-way ANOVA (α = 0.01) and the Tukey’s test (α = 0.05), a post-hoc test that indicates which of the three cycle sets are significantly different.

The overall success of single cell analysis was quantified using the average random match probability (RMP) for the samples across each cycle set using a 100 and 150RFU analytical threshold. The RMP was calculated (without the use of theta) using the average allele frequencies in the updated NIST 1036 U.S. Population Dataset^30^. The Scientific Working Group on DNA Analysis Methods (SWGDAM) recommended “2p” rule was used to account for the effects of allele dropout^31^ and was applied to any locus where a single allele was present, regardless of whether or not this locus was a known homozygote. This provided a generally more conservative estimate of the genotype frequency for the locus in question. Success was further evaluated using threshold-based dropout metrics and inter-locus correlations of signal (Spearman correlation). The correlations were run across the 29, 30, and 31 cycle sets to evaluate the association between the total allelic products among loci. The correlation coefficients were qualitatively labeled using the following verbal scales: very high (0.9–1.0), high (0.7–0.89), moderate (0.4–0.69), low (0.1–0.39), and negligible (0.0–0.1)^32^; correlation coefficients can range from -1 to 1, where a positive value indicates a positive relationship—one value increases the other value increases. Heat maps were created using Morpheus (https://software.broadinstitute.org/morpheus).

### Ethics declarations

This study was approved by the Syracuse University Institutional Review Board, all samples were stripped of identifying information, all experiments were performed in accordance with relevant named guidelines and regulations.

### Informed consent

Informed consent was obtained from all participants and/or their legal guardians. Syracuse University IRB (IRB00000069), project IRB#: 19-180.

## Supplementary Information

Supplementary Information.

## Data Availability

The datasets generated during and/or analyzed during the current study are available from the corresponding author on reasonable request.
